# Novel Hydrophobic Functionalized UiO-66 Series: Synthesis, Characterization, and Evaluation of Their Structural and Physical–Chemical Properties

**DOI:** 10.3390/ijms25010199

**Published:** 2023-12-22

**Authors:** Pilar Narea, Iván Brito, Yurieth Quintero, Esteban Camú

**Affiliations:** 1 Departamento de Química, Facultad de Ciencias Básicas, Universidad de Antofagasta, Campus Coloso, Antofagasta 1240000, Chile; pilar.narea@uantof.cl; 2 Materials Science and Process Engineering Ph.D. Program, Universidad Tecnologica Metropolitana (UTEM), Santiago 8940577, Chile; yurieth.mar@gmail.com; 3 Advanced Mining Technology Center (AMTC), Universidad de Chile, Santiago 8370451, Chile; 4 Departamento de Ingeniería Química y Bioprocesos, Facultad de Ingeniería, Pontificia Universidad Católica de Chile, Santiago 8331150, Chile; esteban.camu@uc.cl

**Keywords:** hydrophobic MOFs, post-synthetic methods, aqueous media, acid media

## Abstract

A novel set of four functionalized hydrophobic UiO-66-NHR series were synthesized through postsynthetic procedures, utilizing various benzoyl chlorides and UiO-66-NH_2_ as starting materials. This synthesis method was carried out by employing p- (1) and o-toluoyl (2), as well as 2- (3) and 4-fluorobenzoyl (4) substituents. The analysis of the resulting compounds was performed using conventional spectroscopic methods such as FT-IR and ^1^H NMR to quantify the conversion rate into amide. Furthermore, SEM and XPS techniques were employed for morphological and surface analysis. Finally, the evaluation of the chemical stability and contact angle using the sessile drop method was performed to evaluate the technological potential of these compounds for application in aqueous and acidic media (such as selective separation of different metals and wastewater recovery).

## 1. Introduction

Metal–organic frameworks have been of attractive interest due to their wide versatility in a variety of research fields [1,2,3,4,5]. The well-thought-out synthesis and the use of proper metal nodes and organic ligands allow for the design of materials with tunable porosity, high surface areas, and great variability and shapes [6,7]. These features show that MOF-based materials are promising for a handful of applications in a wide range of technologies, including gas uptake and separation [8], gas storage [9], electrochemical applications [10], sensors [11], and water treatment [12]. This last focus has been of interest among several research groups around the world that have studied the syntheses and applications of hydrophobic MOFs for different aqueous sources for different applications such as ion isolation [13], detection and elimination of pollutants [14], heavy metal remotion [15], water treatment and regeneration, as well as other environmental applications [16].

MOFs are compounds of great interest in applications due to their crystalline, porous structure, but above all because of their easy design, which allows their structure to be adapted according to the properties to be studied, obtaining varied morphologies thanks to the presence of metal ions and various ligands [17].

MOFs are also known as porous coordination polymers (PCPs) due to their large surface area, high porosity, controllable pore sizes, and abundant adsorption sites, which make them potential materials for applications such as wastewater treatment involving the elimination of toxic metallic contaminants and organic contaminants [12,18,19,20,21]. Their modular structures can be used as adsorbents that interact with the adsorbate through interactions such as hydrogen bonding, π–π, acid–base interaction (ABI), coordination (Lewis ABI), and hydrophobic interaction [22]. They can also function as additives in semipermeable membranes for water recovery [18,23,24,25,26].

These compounds, when in contact with an aqueous matrix, must have hydrostability so that they do not degrade and dissolve in water. In aqueous media, water molecules usually easily attack the bonds between the metal ion and the organic ligand, leading to the structural collapse of the MOF or the change in the crystalline phase through the decomposition or displacement of the ligands through the hydrolysis reaction [27]. Therefore, it is crucial to decrease the interactions between water and the MOF structure to avoid affecting its structure [28].

In general, MOFs must be stable under environmental conditions. This can be a decisive factor for their usability in water and/or wastewater applications. However, the MOFs instability in extreme environmental acid conditions (pH 0–2) drives their shift and decomposition due to hydrolysis reactions [27,29]. For instance, a Cu-based metal–organic framework (MOF) called HKUST-1 (from the Hong Kong University of Science and Technology), synthesized from copper nitrate and trimesic acid (TMA), is a good example of high porosity and thermal stability [30]; however, it is very susceptible to moisture, restraining its use in water treatment applications [31].

In this sense, UiO-66 compounds are the most feasible candidates for structural modifications, integrating or transforming organic groups within the structure [21,32]. For example, the functionalization of UiO-66-NH_2_ with isostearic acid has been used to increase the hydrophobicity and enhance the Rhodamine 6G (Rh 6G) adsorption affinity in an aqueous medium [33]. The postsynthetic modification (PSM) has been converted into a useful tool to variate and enhance the MOF’s properties according to the proposed application.

MOFs with amino groups within the structure can react using PSM in an easier way to generate amide-functionalized MOFs [21,32,34,35], which can contain functional groups within the structure with low surface energy, modifying the physical hydrophilic properties of the resulting compound into hydrophobic, making the modified MOFs suitable for use in aqueous sources.

In an attempt to generate compounds with good hydrophobicity, a series of hydrophobic MOFs were synthesized via the PSM method, introducing an aromatic moiety with substituents of low surface energy such as methyl (-CH_3_) and fluoro (-F) groups in ortho and para positions in the aromatic moiety. The synthesized compounds could enhance moisture resistance and modify MOF hydrophobicity. The substituent effect and the influence of hydrophobicity according to the substituent’s position in the aromatic ring moiety were achieved in two different MOF systems obtained in this work.

## 2. Results and Discussion

### 2.1. Syntheses

**Figure 1:**
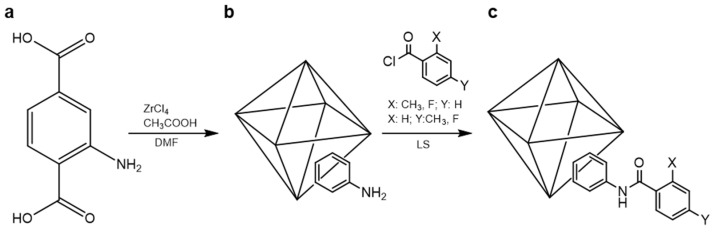
Synthetic pathway of amide-functionalized UiO-66-NHR. (**a**) Free ligand used in the MOF precursor; (**b**) schematic Metal-Oxide cluster within UiO-66-NH_2_ and (**c**) functionalized UiO-66-NHR. After post-synthesis.

To achieve a hydrophobic focus in the compound candidates, UiO-66-NH_2_ was functionalized using the following benzoyl chlorides: p- and o-toluoyl chlorides, as well as 4- and 2-fluorobenzoyl chlorides, resulting in compounds (**1**–**4**), respectively (see Figure 1 for more details). The experimental conditions were under solvent-free methods, with the aim of generating eco-friendly and low-cost approaches to these types of compounds. At the beginning, the UiO-66-NH_2_ precursor is a hydrophilic compound due to the great possibility of generating hydrogen bonds with some key parts of the structure, such as the amino group and/or carboxylate oxygen. The use of the functionalized benzoyl chlorides to generate the respective hydrophobic compounds via amide formation is an easy-to-deal-with way to transform the hydrophilic precursor into a hydrophobic one due to the amide functionalization blocking the water solvation, resulting in an important decrease in hydrogen bond interactions.

The presence of the functionalized amide compound promotes stability to water and moisture, which is a key feature for distillation membrane (DM) technologies [23,25,26,36]. To achieve successful functionalization, one important factor is that the MOF compounds have a reactive position, and there is no steric hindrance with the substituents as well.

Finally, the isolated compounds are air, moisture, and a thermally stable pale-yellow microcrystalline solid. The composition analyses and, likewise, other characterization experiments are consistent with the proposed structures (see section below for more details).

### 2.2. Infrared Spectra

**Figure 2:**
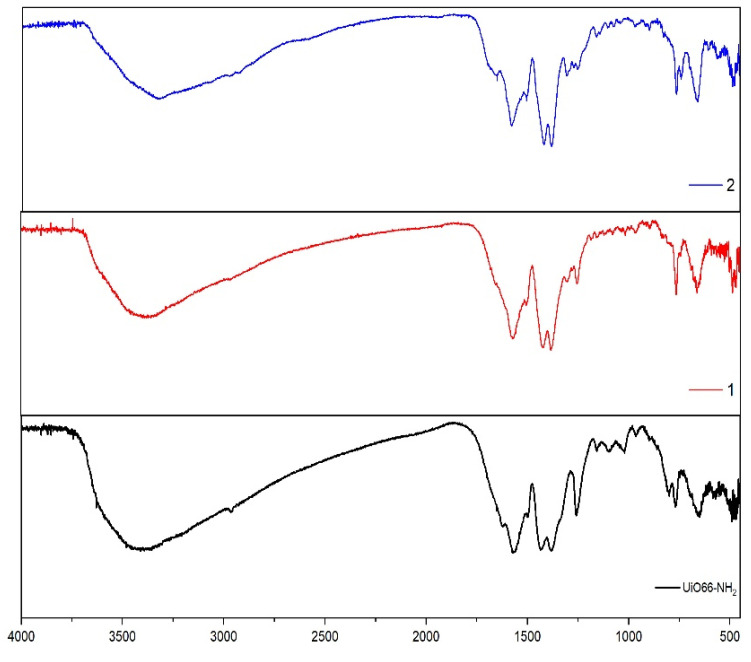
FT-IR spectra of UiO-66-NH_2_ (black line) and compounds (**1**) (red line) and (**2**) (blue line).

**Figure 3:**
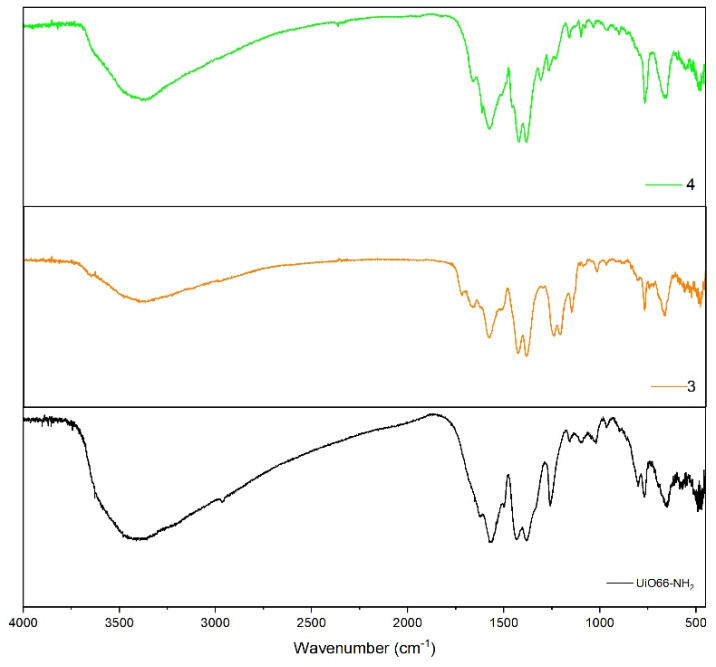
FT-IR spectra of UiO-66-NH_2_ (black line) and compounds (**3**) (orange line) and (**4**) (green line).

The solid-state FT-IR spectra of the compounds (**1**–**4**) show a characteristic pattern that fits with the proposed structures (see Figure 2 and Figure 3). For instance, the absorption bands observed at 3436 and −3330 cm^−1^ show the presence of the -NH_2_ group in the UiO-66-NH_2_ precursor (asymmetric and symmetrical vibration). As a first proof of the successful functionalization of compounds **1**–**4,** the decrease in these bands shows just one, due to the formation of UiO-66-NHR. In these spectra, it is quite difficult to identify this because, in the same region (3200–3040 cm^−1^), a wide band appears, generating a partial overlapping of the -NH_2_ bands. The wide bands that appear in the region over 3000 cm^−1^ are the -OH vibration band, which can be attributed to the -OH terminal groups of the Zr_6_O_4_(OH)_4_ cluster. However, if UiO-66-NH_2_ and the hydrophobic resulting compounds are compared, it is possible to observe a downshift in the region 1500–1300 to 1300–1270 cm^−1^, attributable to the stretching C-N vibration [37].

Another interesting proof of the successful functionalization and stability of the precursor in the reaction conditions is the specific pattern of each substituent of compound (**1**–**4**), respectively. For instance, in the band close to 750 cm^−1^ in compounds (**1**) and (**4**), we can observe a band corresponding to =C-H bending of the p-substituted ring [37]. Additionally, in compounds (**3**) and (**4**), we can observe a band close to 1160–1148 cm^−1^, corresponding to aromatic ring C-F vibration [37].

### 2.3. NMR Spectroscopy

**Figure 4:**
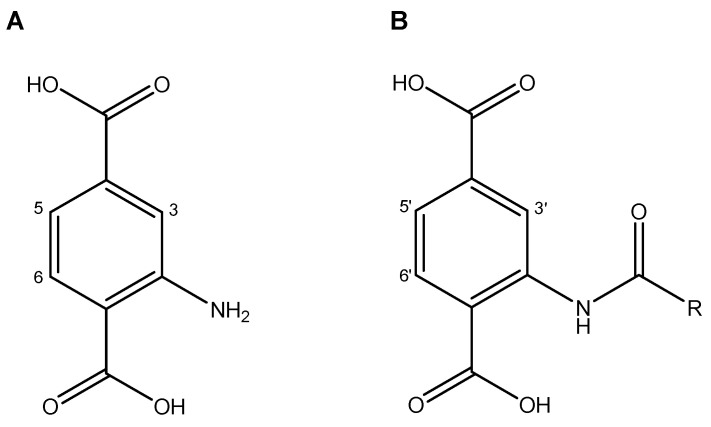
C-H assignments in organic linker for NMR spectra. (**A**) precursor linker and (**B**) post-synthetic amide functionalized linker.

**Figure 5:**
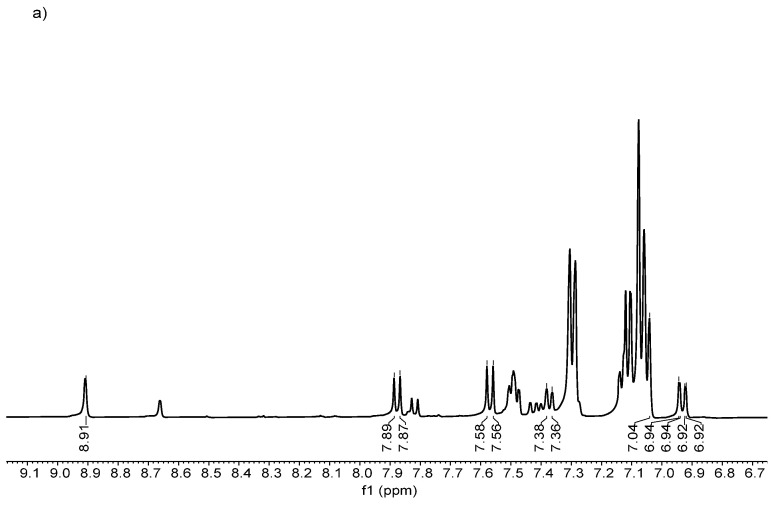

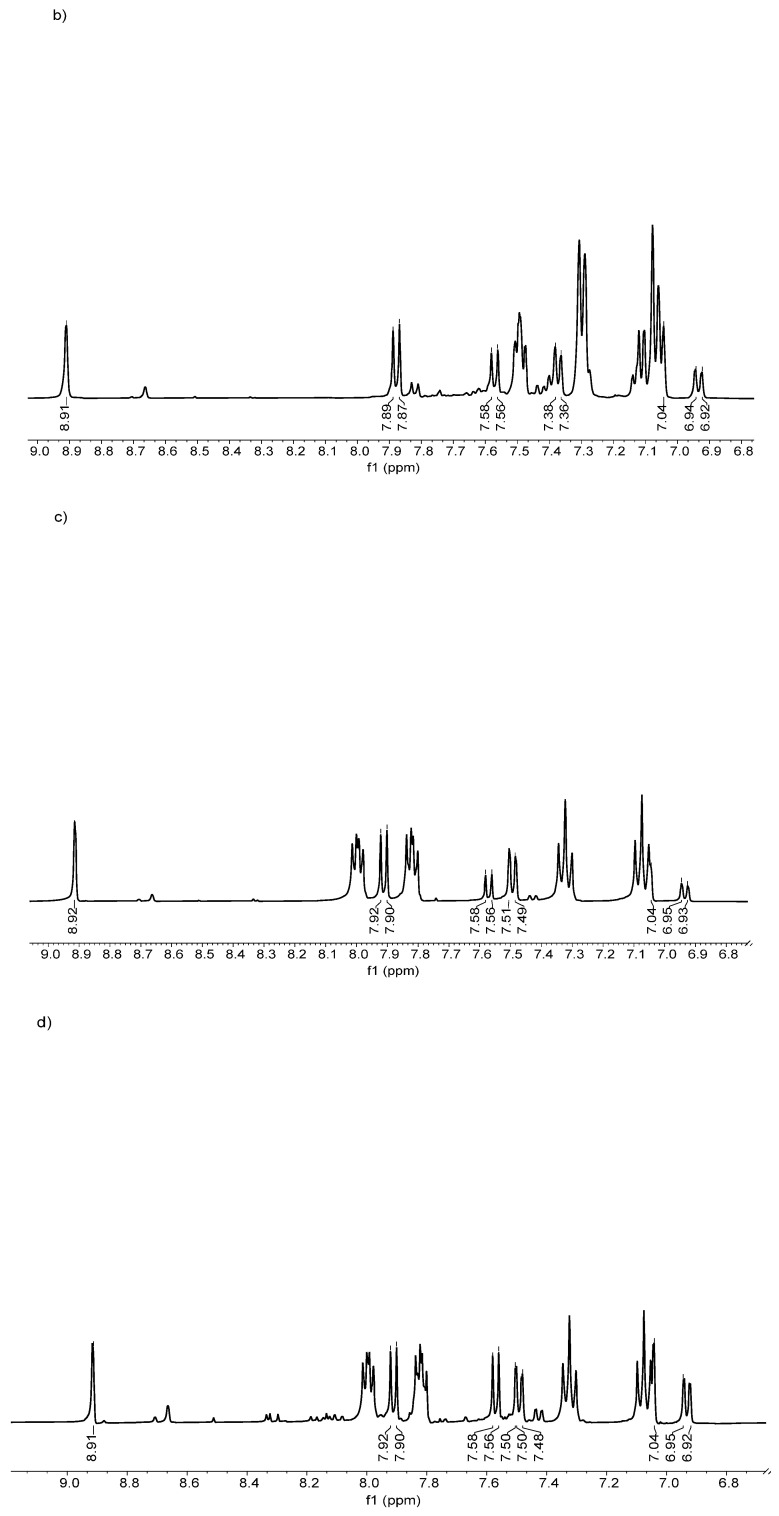
^1^H NMR spectra of (**a**) (**1**), (**b**) (**2**), (**c**) (**3**), and (**d**) (**4**).

Nuclear magnetic resonance (NMR) was carried out on the resulting compound and compared with the UiO-66-NH_2_ (see Figure S1) precursor to quantify the conversion ratio into amide after PSM. The results are given in Table 1. For this purpose, the samples were digested via a reported method using a CsF/D_2_O/DMSO-d_6_ mixture [35]. The conversion ratio from amine to amide functionalization was calculated using the under-peak area integration related to the proton in C-3 of the UiO-66-NH_2_ precursor ligand (see Figure 4).All spectra are depicted in Figure 5.

In general, the conversion ratio confirmed that the resulting compound had a moderate postsynthetic yield of over 50%. Concerning the conversion percentage, a plausible explanation is the substituent effect of the fluoro in different positions in the aromatic ring, due to the ortho-position-related carbonyl group positively affecting the electron density, making the chlorine atom a better-leaving group, translating to a yield close to 74% in the compound (**4**).

However, due to the para position in the fluoro substituent in compound (**3**), it is not able to generate the same electronic effect. In the case of compound (**2**), the presence of a p-methyl group favors the electronic effect of the chlorine atom as a leaving group; meanwhile, the o-methyl group does not have the same effect considering the withdrawing and donor electronic nature of fluoro and methyl substituents, respectively [38].

### 2.4. Crystallographic Studies

**Figure 6:**
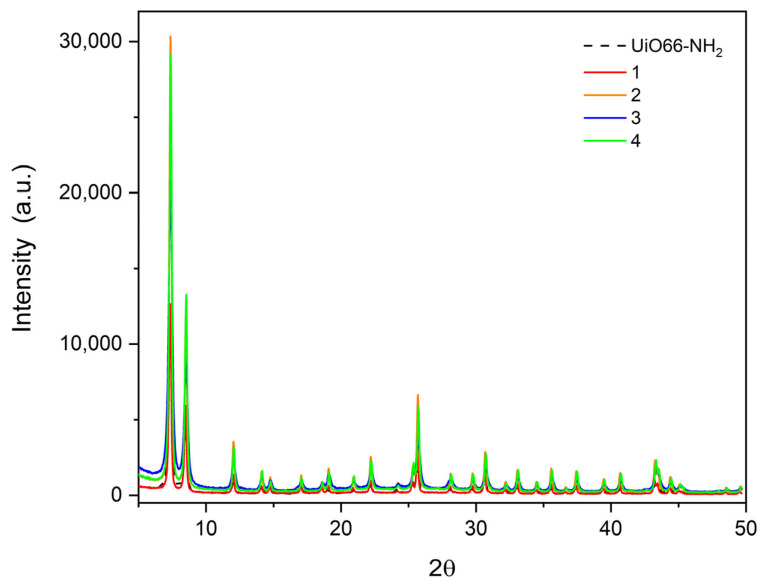
Powder XRD Patterns of UiO-66-NH_2_ (dashed line) and hydrophobic compounds (**1**–**4**).

The powder X-ray diffractogram of UiO-66-NH_2_ compared with the resulting compounds is depicted in Figure 6. The pattern of the synthesized UiO-66-NH_2_ fits well with the reference previously reported in the literature [39]. The intensity and the well-shaped peaks show the high crystallinity of the synthesized compounds (**1**–**4**). For instance, the peaks appearing in 7.357, 8.499, and 25.68° are quite identical to those reported for the UiO-66-NH_2_ precursor. It is possible to conclude that the benzoyl-functionalized amides generated from PSM do not generate significant changes in the cell structure, retaining the crystallinity of the resulting compounds.

**Figure 7:**
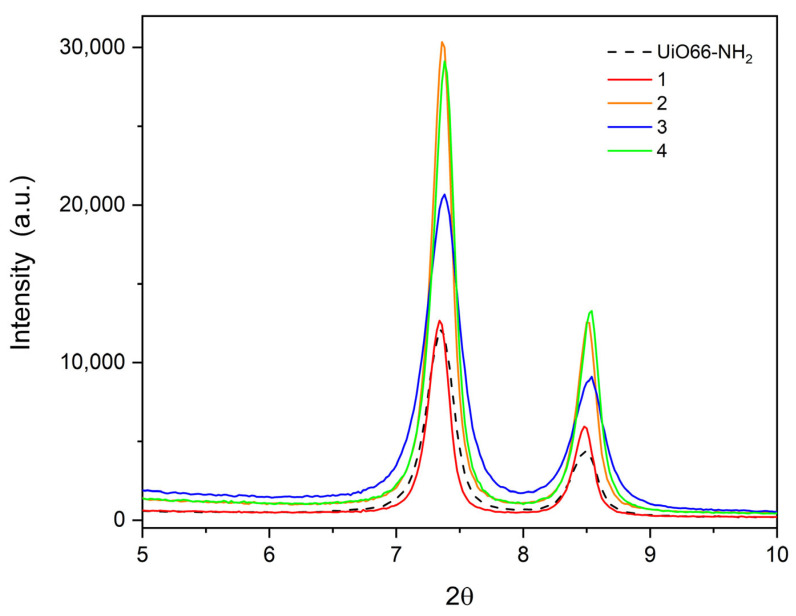
Major peaks in compounds (**1**–**4**).

Another interesting aspect is the difference in the main peaks in each compound, which is due to the addition of more atoms and the electronic scattering of the atoms inside the unit cell. For example, in compounds (**2**) and (**4**), the peaks close to 6–10° are more intense than compounds (**1**) and (**3**), and these are more intense than UiO-66-NH_2_. This is due to the substituent effect in the resulting compounds, where in compounds (**2**) and (**4**) they are more scattered than in compounds (**1**) and (**3**) (see Figure 7). This is correlated with the crystallite (grain) size calculated with Scherrer’s equation [40] (Table 2), which shows an average size of ~25–30 nm, which is in agreement with the information given by SEM measurements (see Section 3.6 for more details).

### 2.5. XPS Spectroscopy

**Figure 8:**
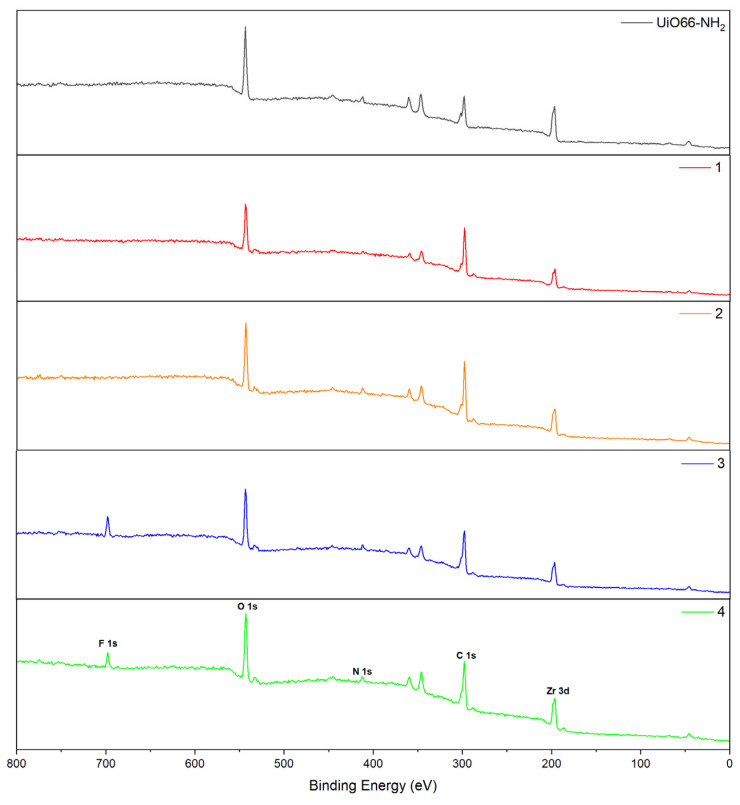
Survey region XPS spectra of the synthesized compounds.

The chemical states of UiO-66-NH_2_ and compounds (**1**–**4**) were analyzed using XPS techniques and are depicted in Figure 8. The survey spectra of all compounds show characteristic peaks of Zr 3d, C 1s, N 1s, and O 1s. Additionally, in compounds (**3**) and (**4**), the F 1*s* is also observed in each spectrum, showing a successful postsynthetic treatment. It is interesting to highlight the appearance of two low-intensity peaks close to 520 eV and 275 eV in compounds **1**–**4** corresponding to amide groups, corresponding to O 1s → π* (N-(C=O*)-C) and C1*s* → π* (N-(C*=O)-C), respectively [41].

On the other hand, the deconvoluted high-resolution peaks of the UiO-66-CONH_2_ reference show all the characteristic peaks corresponding to the organic ligand, in good agreement with the pattern reported previously in the literature [42]. Additionally, a peak close to 288 eV is observed in all functionalized compounds (**1**–**4**). The peak corresponds to the -NH(C=O) bond, according to what was previously reported [43], which is complemented by the peak appearing close to 530 eV in the O 1s region belonging to N-(C=O)-C (aromatic bond) [44]. For compounds (**3**) and (**4**), the peaks close to 685 eV attributed to F 1*s*, specifically to the aromatic C—F bond, are also observed.

Semiquantitative analysis was also performed, with the aim of evaluating the amine conversion ratio into amide in the resulting compounds to establish the relationship between hydrophobic behavior and the conversion ratio (see Section 3.3).

According to XPS analysis, all compounds show a moderate conversion ratio, as proof of the solvent-free reaction (see Figure S2). Additionally, the semiquantitative analysis shows that the conversion ratios in each compound were around 28 to 63% (see Table 3 for more details), which agrees with the evaluation of their hydrophobic properties (see Section 3.3 for more details).

### 2.6. Scanning Electron Microscopy

**Figure 9:**
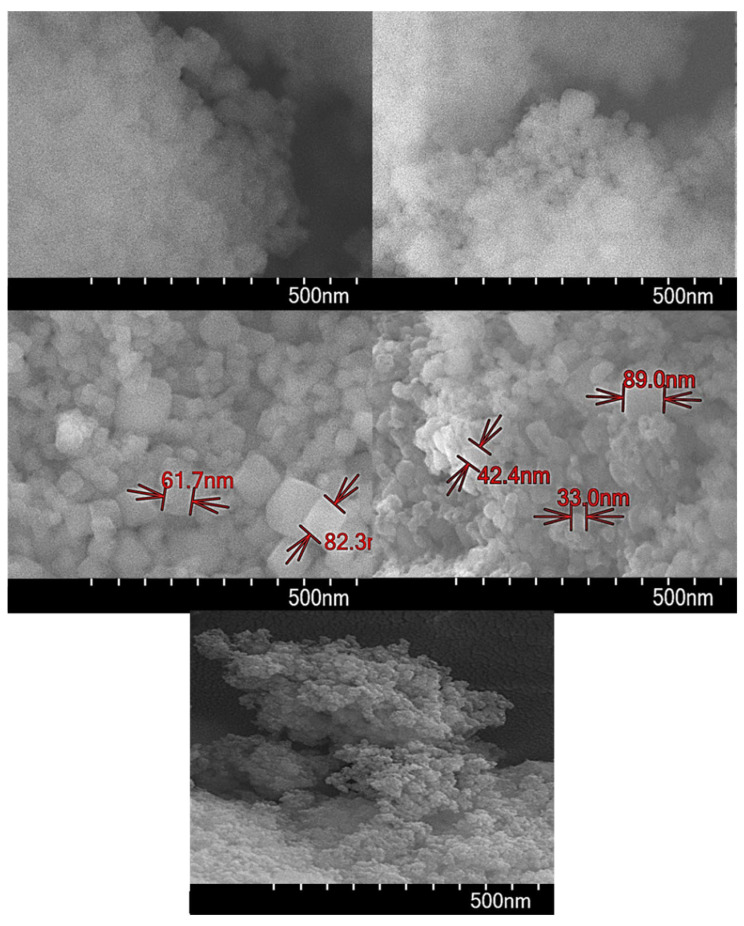
SEM images of the resulting compounds at 500 nm.

SEM measurements show that all compounds have a particle size below 100 nm (see Figure 9 for more details). Concerning the surface texture, it is possible to observe a plain surface with conglomerates built by little cubic-shaped nanocrystals of the resulting MOFs.

On the surface of the matrix, a homogeneous distribution of the compound can be observed, which suggests that the sample exhibits a high level of purity. According to the images obtained through SEM-EDX analysis reported in Figure S3, it verifies the high purity of the sample, as the surface composition analysis of the compounds contains the elements belonging to the proposed MOFs distributed uniformly within the matrix.

### 2.7. Thermal Stability

The thermal behavior of compounds (**1**–**4**) was performed to understand the stability of the resulting compounds and their possibilities in the use of modified membranes with these compounds for the distillation membrane process a posteriori. For this purpose, the compounds were heated up to 400 °C at a heating rate of 10 °C min^−1^ under nitrogen flow at 20 mL min^−1^.

**Figure 10:**
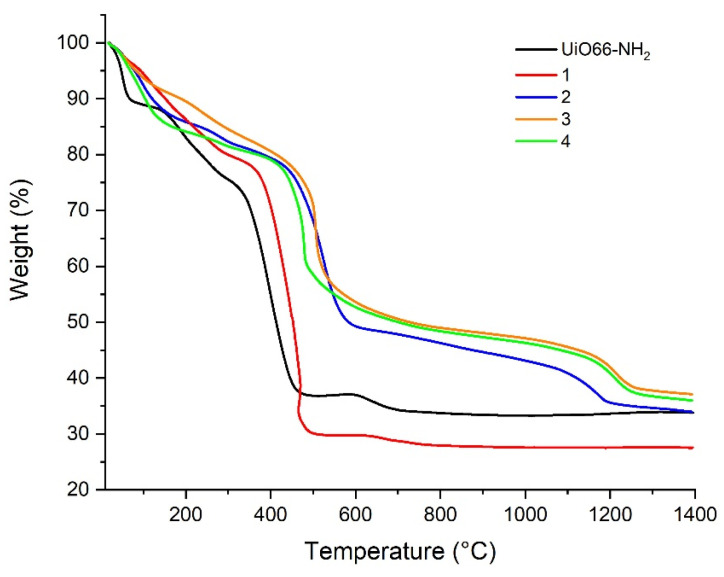
TGA curves of the resulting compounds compared with UiO-66-NH_2_.

In general, the resulting compounds are very stable until 120 °C. The thermal processes are similar for all compounds. Compared with the UiO-66-NH_2_ precursor, the thermal stability of compounds (**1**–**4**) is quite similar between them, showing the following order in their thermal stability: UiO-66-NH_2_ < (**1**) < (**4**) < (**2**) ≈ (**3**). It can be concluded that this thermal behavior has a relationship with the yield increase in the postsynthetic treatment compared with UiO-66-NH_2_ (see Figure 10 for more details). Additionally, the enhancement of thermal stability is related to the yield of each compound after postsynthetic treatment.

On the other hand, the loss weight under 100 °C is related to the desorption of solvent molecules such as MeOH or H_2_O and/or moisture from the porous surface of compounds. The nonreplaced DMF on the pore surface is lost between 100 and 300 °C for UiO-66-NH_2_ and compound (**1**). Between 150 and 430 °C for compounds (**2**), (**3**), and (**4**), the loss weight is related to the Zr_6_O_4_(OH)_4_ cluster dehydration becoming Zr_6_O_6_. The decomposition of the inorganic cluster into ZrO_2_ is observed around 300 °C. However, this step is not preceded by an apparent plateau [45], which is indicative that the decomposition process starts before complete solvent decomposition.

### 2.8. Chemical Stability

**Figure 11:**
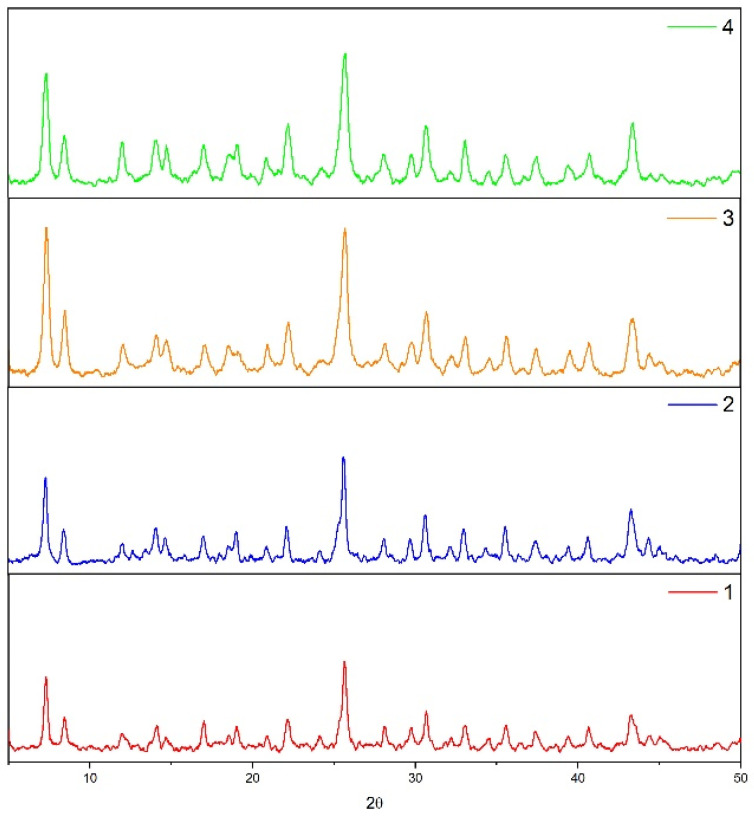
XRD Pattern of postexposed compounds in acidic medium.

The synthesized compounds were subjected to chemical stress, leaving the samples in a water and sulfuric acid solution of 0.01 M for 96 h to determine their chemical stability under these experimental conditions, which were then analyzed using powder XRD after chemical exposition (see Figure 11). Surprisingly, all compounds maintain the same structural parameters, according to the results shown in powder XRD, remaining with an unaltered structure under these conditions. This last is a good proof to demonstrate the high chemical and structural stability of the resulting compounds in similar conditions as acid effluents.

It is important to notice that the Zr_6_O_4_(OH)_4_ cluster is a more stable secondary building unit in the MOF’s development [46]. This is due to the high charge density, which can polarize the O atoms of the carboxylate moiety within the structure, generating strongbonds of the Zr-O type. Moreover, the good stability is also attributed to the presence of low surface energy groups, which can combine the hydrophobic–electronic effect.

### 2.9. BET Isotherms

**Figure 12:**
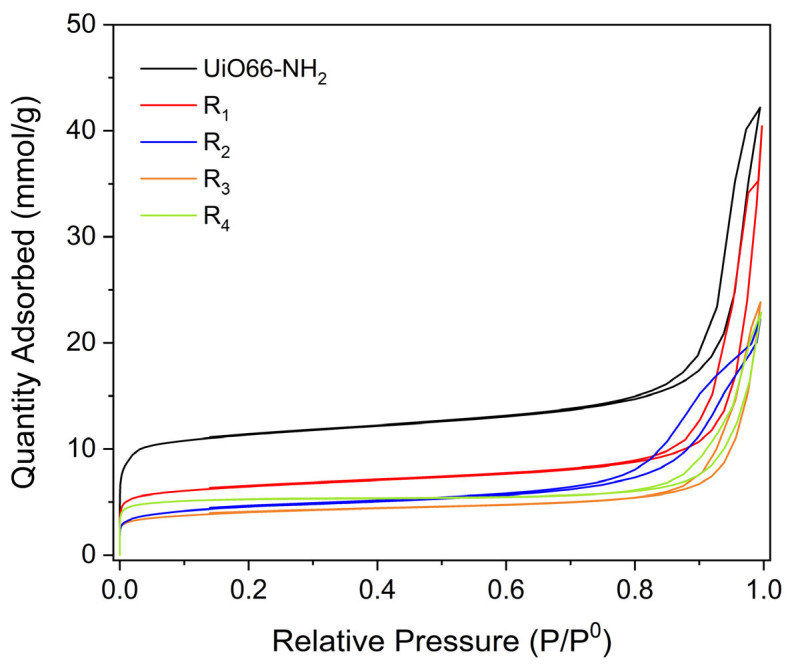
N_2_ adsorption–desorption isotherms of the different samples at −196 °C.

Figure 12 shows the nitrogen adsorption–desorption isotherms for UiO-66-NH_2_ and compounds (**1**–**4**). All the samples showed a type IV isotherm, related to mesoporous materials, with an H3 hysteresis cycle, related to platelike pores. However, at low relative pressure, we can observe the presence of micropores in the samples.

The BET-specific area obtained for UiO-66-NH_2_ was 984 m^2^ g^−1^, which agrees with previous reports [47]. Table 4 shows the specific area (S_BET_), micropore volume (Vm), and average pore diameter (Dp) for the samples studied.

As it can be seen, the PSM generates a decrease in the specific area and micropore volume of the samples; this might be due to the blockage of the pores for the incorporation of the substituted benzoyl. However, this PSM does not affect the average pore diameter of the materials. This decrease in the specific area is related to the decrease in the band located at 3436 and −3330 cm^−1^ obtained using FT-IR, since the incorporation of more bulky groups affects the interaction of nitrogen molecules with the samples.

### 2.10. Contact Angle Evaluation

**Figure 13:**
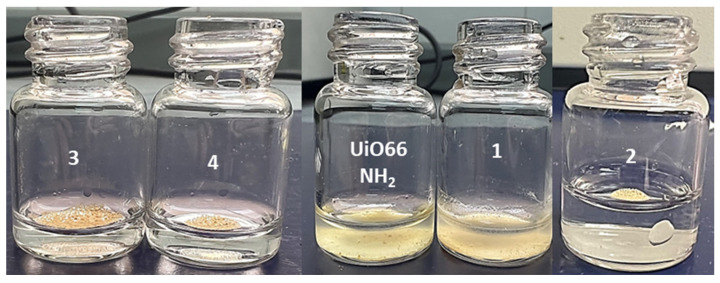
Hydrophobic behavior of compounds (**1**–**4**).

As a qualitative approach to the hydrophobicity of the resulting compounds compared with UiO-66-NH_2_, a test was performed, pouring a small amount of the compound into a flask with water (Figure 13). The test showed that compounds (**2**–**4**) rested above the water meniscus; meanwhile, compound (**1**) fell to the bottom of the flask due to the more hydrophilic contacts and low postsynthetic conversion.

A qualitative analysis was carried out to observe the hydrophobicity of the synthesized compounds. The compound was added over an amount of water, where compound (**1**) shows the less-spherical drop when the test was performed (see Figure S4).

Considering the qualitative test, contact angle studies were carried out using the sessile drop method (see Figure S5), where compound 1 showed the lowest contact angle in the recorded measurement according to the standard values reported previously in the literature [48]. The values shown in Table 5 are indicative of the fact that the hydrophobicity of UiO66-NH_2_ can be tuned via postsynthetic methods. The tunable properties are derived from the low surface energy groups present in the amide backbone. For compound (**1**), a contact angle less than 70° is obtained, making it more hydrophilic than compounds (**2**), (**3**), and (**4**), which show hydrophobic characteristics with a contact angle greater than 100°.

Analyzing the substituent characteristics and their position in the aromatic ring, it should be expected that the compounds modified with the -F groups present a greater contact angle than the compounds substituted by the methyl groups because this group has less surface energy than the -CH_3_ group.

Concerning the group positions in the aromatic ring, the ortho position with respect to the amide group presents greater hydrophobicity. A plausible explanation is that the presence of these groups prevents the water molecules from generating intermolecular interactions with the groups that have N and O atoms in their structure.

The contact angle results showed that the compound (**3**), which has a fluorine group in the ortho position to the amide group, has the highest contact angle, followed by compound (**4**). Compound (**2**) generated the third hydrophobic compound, which has a -CH_3_ group in the para position of the aromatic ring.

The results obtained suggest that the hydrophobicity of the modified compounds is closely related to the post-synthetic yield percentage shown in the previous NMR analysis, which shows that compounds with a 50% modification can present this feature. Therefore, the decrease in hydrophobicity compared to the initial compound indicates that post-synthesis occurred in all four but that the percentage of substitution within the structure for (**1**) was not sufficient to generate the required hydrophobic properties.

## 3. Materials and Methods

### 3.1. Material Source

Solvents and reagents were handled without further purification and according to the standard procedures described in the literature [49]. Reactions were carried out under standard procedures. All reagents were analytical-grade materials that were purchased from Sigma-Aldrich (St. Louis, MO, USA).

### 3.2. General Synthetic Procedure

#### 3.2.1. Synthesis of UiO-66-NH_2_

**Scheme 1:**
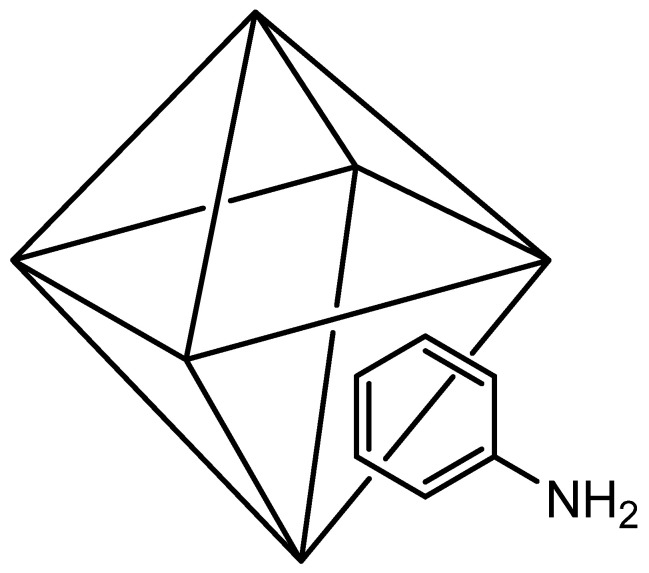
Octahedron cluster of UiO-66-NH_2_.

UiO66-NH_2_ (Scheme 1) was carried out following the procedure reported by Schaate et al. [50] with slight modifications, using 280 mg of ZrCl_4_ (1.2 mmol) dissolved in 36 mL of DMF with 30 eq. of acetic acid.

On the other hand, 216 mg of NH_2_-bdc (1.2 mmol) was also dissolved in 36 mL of DMF with 4 eq. of distilled water (4.8 mmol). Then, both solutions were charged in a round-bottom flask and sonicated for 20 min at 60 °C. The resulting suspension was poured into a beaker, treated under solvothermal conditions for 24 h at 120 °C, and cooled to room temperature, obtaining a pale-yellow solid that was washed with DMF (24 h) at room temperature and centrifugated to isolate the resulting compound. Finally, the solid was washed with MeOH (3 times); the last wash was performed for 24 h at room temperature.

#### 3.2.2. Post-Synthetic General Procedure of UiO-66-NHR

**Scheme 2:**
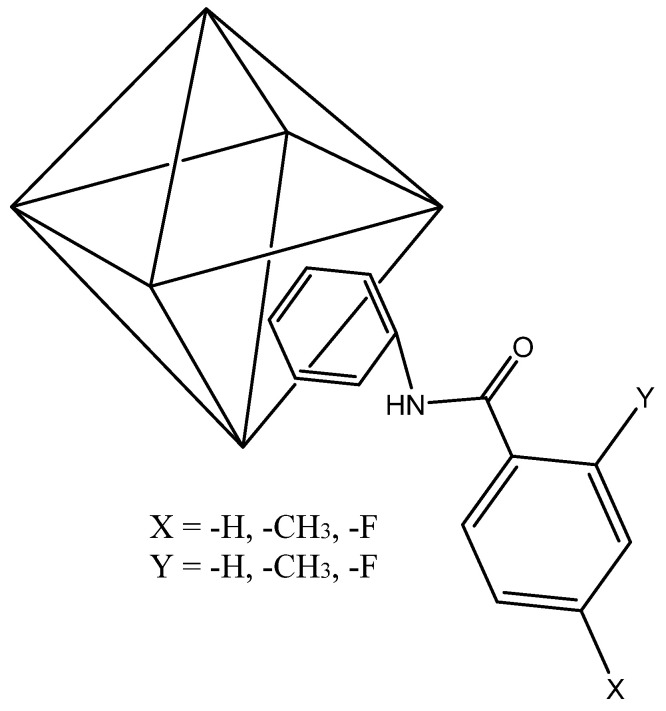
Octahedron cluster of hydrophobic UiO-66-NHR: (**1**) X = (-CH_3_), Y = (-H); (**2**) X = (-H), Y = (-CH_3_); (**3**) X = (-F), Y = (-H); (**4**) X = (-H), Y = (-F).

Hydrophobic UiO-66-NHR (see Scheme 2 for more details) was synthesized under solvent-free conditions using the procedure reported by Hintz et al. [35], using 500 mg of solid UiO-66-NH_2_ slowly stirred in a round bottom flask at 115 °C. Then, 10 mL of substituted benzoyl chlorides were added dropwise, slowly, and stirred for 20 min.

Finally, the solids were filtered and washed several times with acetone.

### 3.3. Characterization

Solid-state FT-IR spectra were recorded on a Thermo Electron Corporation model Nicolet Avatar 330 spectrophotometer with KBr disks in the 4000 to 400 cm^−1^ range. NMR spectra were recorded at 298 K with a Bruker Ascend TM at 400 MHz. ^1^H NMR spectra are reported in parts per million (ppm, d) relative to tetramethylsilane (Me_4_Si), with the residual solvent proton resonances used as internal standards. To measure the ^1^H NMR of hydrophobic UiO-66-NHR in solution, the digestion of the resulting compounds was performed using cesium fluoride (CsF) in a mixture of D_2_O/DMSO-d_6_, using UiO-66-NH_2_ for comparative purposes, according to the procedure previously reported in the literature [35]. The X-ray powder diffraction data of (**1**–**4**) were collected at room temperature using a Bruker D8 Discover automated diffractometer (Bruker, Billerica, MA, USA) in Bragg–Brentano geometry, using CuKα_1_ radiation (λ = 1.54177 Å). The samples were scanned in the range of 2θ = 2–50° with a step size of 0.026 °s^−1^ per step. To check the structural parameters, a Rietveld refinement [51] was carried out using the Fullprof program, version 7.95 [52]. The chemical stats of the prepared films were examined using X-ray photoelectron spectroscopy (XPS). Measurements were carried out at room temperature on a surface analysis station 150, BRUKER RQ300/2, using the Al Kα line (1486.6 eV) as the excitation source. Survey spectra were acquired at pass energies of 80 eV (energy resolution of 0.87 eV). Data analysis was performed using CasaXPS software, version 2.3.25PR1.0 [53]. The binding energy for the C1s hydrocarbon peak was set at 284.8 eV in the calibration procedure. The core level spectra were acquired with an energy step of 0.1 eV and using a constant pass energy mode of 20 eV (energy resolution of 0.48 eV).

### 3.4. Scanning Electron Microscopy (SEM)

The morphology of the samples and their chemical composition were analyzed using a Hitachi SU-5000 FESEM (Hitachi Ltd., Tokyo, Japan), which was equipped with an energy dispersive spectrometer with an XFlash6I30 Bruker detector (Bruker, Billerica, MA, USA).

### 3.5. Thermal Stability Studies

Thermogravimetry analyses (TGA) were carried out on an STA 448 Jupiter F3-type simultaneous thermal analyzer (Netzsch, Selb, Germany). For TGA, 5.0 mg of the samples were used as microcrystalline powders. The used sample cells were aluminum oxide pans. The parent reagents were heated up to 400 °C at a heating rate of 10 °C min^−1^ under nitrogen flow at 20 mL min^−1^.

### 3.6. Chemical Stability Studies

The chemical stability of the resulting compounds was measured in an acidic medium using sulfuric acid (H_2_SO_4_) at pH = 2 for 96 h with constant stirring. To evaluate the status of the exposed MOFs, powder XRD analyses were carried out before and after acid exposure.

### 3.7. BET Isotherm Studies

The determination of the specific area (S_BET_) for each sample was carried out in a Micromeritics 3FLEX apparatus for volumetric nitrogen adsorption–desorption, using 0.15–0.30 g of each sample, which were degassed at 180 °C for 18 h, reaching a final pressure of 1 × 10^−3^ mmHg. Subsequently, the analysis was performed in a glass sampling cell at −196 °C, and the S_BET_ calculations were performed using the Rouquerol criteria.

### 3.8. Contact Angle Studies

Contact angle measurements to evaluate the hydrophilicity of the synthesized compounds were determined by sessile drop methods using an optical tensiometer from biolin scientific, model Attension theta, at room temperature over a period of 0 to 60 s with precisely 4 μL of water. Still images of the contact angle at different intervals were captured with a high-speed camera, and the reported results are the average values of five samples.

## 4. Conclusions

Solvent-free PSM methods were achieved successfully, resulting in four hydrophobic UiO-66-NHR compounds. The conversion percentage post-synthetic and the positional effects of the used substituent were found to be key factors in enhancing the hydrophobicity of superhydrophobic compounds, taking this work as a checkpoint according to contact angle and qualitative measurements against water. The use of a fluoro substituent is the better method to enhance hydrophobicity under mild conditions, probably due to the low surface energy compared with the methyl group, which disfavors hydrogen bond interactions. The thermal and chemical stability of these materials may make them good candidates for use in aqueous applications as adsorbents for environmental remediation or as additives in membrane technologies for water recovery, such as membrane distillation.

## Figures and Tables

**Table 1:** Conversion ratio in compounds (**1**–**4**) treated via PSM.

Compound	Ratio	Conversion (%)
(**1**)	0.44	30.79
(**2**)	1.15	53.40
(**3**)	2.72	73.10
(**4**)	1.04	51.07

**Table 2:** Cristallite grain size of compounds (**1**–**4**).

Compound	Peak Position (2θ)	FWHM (2θ)	Crystallite Size (nm)
UiO-66-NH2	7.345	0.2815	29.55
(**1**)	7.334	0.2140	38.86
(**2**)	7.357	0.2158	38.54
(**3**)	7.366	0.3312	25.11
(**4**)	7.373	0.2310	36.01

**Table 3:** XPS amine/amide ratio in compounds (**1**–**4**) treated via PSM.

Chemical Specie	NH_2_ Conc. (%)	HN(C=O) Conc. (%)
(**1**)	71.19	28.81
(**2**)	40.78	59.22
(**3**)	37.46	62.53
(**4**)	43.45	56.55

**Table 4:** Textural properties for compounds (**1**–**4**).

Compound	S_BET_ (m^2^ g^−1^)	Vm (cm^3^ g^−1^)	Dp (nm)
UiO-66-NH2	984	0.30	0.82
**1**	542	0.16	0.81
**2**	330	0.10	0.81
**3**	295	0.09	0.81
**4**	416	0.16	0.81

**Table 5:** Contact angle for compounds (**1**–**4**).

Compound	Contact Angle (°)
UiO-66-NH_2_	32.7
**1**	60.4 ± 2.83
**2**	113.7 ± 1.19
**3**	126.4 ± 2.78
**4**	120.4 ± 2.67

## Data Availability

Data are contained within the article and Supplementary Materials.

## Supplementary Materials

The following supporting information can be downloaded at: https://www.mdpi.com/article/10.3390/ijms25010199/s1.

## References

[1] Meng J., Liu X., Niu C., Pang Q., Li J., Liu F., Liu Z., Mai L. (2020). Advances in Metal–Organic Framework Coatings: Versatile Synthesis and Broad Applications. Chem. Soc. Rev..

[2] Chen L., Xu Q. (2019). Metal-Organic Framework Composites for Catalysis. Matter.

[3] Păun C., Motelică L., Ficai D., Ficai A., Andronescu E. (2023). Metal-Organic Frameworks: Versatile Platforms for Biomedical Innovations. Materials.

[4] Wang C., Liu D., Lin W. (2013). Metal–Organic Frameworks as A Tunable Platform for Designing Functional Molecular Materials. J. Am. Chem. Soc..

[5] Cedrún-Morales M., Ceballos M., Polo E., Del Pino P., Pelaz B. (2023). Nanosized Metal–Organic Frameworks as Unique Platforms for Bioapplications. Chem. Commun..

[6] Zhang W., Taheri-Ledari R., Saeidirad M., Qazi F.S., Kashtiaray A., Ganjali F., Tian Y., Maleki A. (2022). Regulation of Porosity in MOFs: A Review on Tunable Scaffolds and Related Effects and Advances in Different Applications. J. Environ. Chem. Eng..

[7] Garai B., Bon V., Krause S., Schwotzer F., Gerlach M., Senkovska I., Kaskel S. (2020). Tunable Flexibility and Porosity of the Metal–Organic Framework DUT-49 through Postsynthetic Metal Exchange. Chem. Mater..

[8] Wang B., Zhang X., Huang H., Zhang Z., Yildirim T., Zhou W., Xiang S., Chen B. (2021). A Microporous Aluminum-Based Metal-Organic Framework for High Methane, Hydrogen, and Carbon Dioxide Storage. Nano Res..

[9] Li H., Li L., Lin R.-B., Zhou W., Zhang Z., Xiang S., Chen B. (2019). Porous Metal-Organic Frameworks for Gas Storage and Separation: Status and Challenges. EnergyChem.

[10] Dave S., Sahu R., Tripathy B.C. (2022). Electrochemical Applications of Metal-Organic Frameworks.

[11] Gao W., Liu F., Pan C.-W., Zhang X.-M., Liu J.-P., Gao Q.-Y. (2019). A Stable Anionic Metal–Organic Framework with Open Coordinated Sites: Selective Separation toward Cationic Dyes and Sensing Properties. CrystEngComm.

[12] Liu X., Shan Y., Zhang S., Kong Q., Pang H. (2023). Application of Metal Organic Framework in Wastewater Treatment. Green Energy Environ..

[13] Goyal P., Tiwary C.S., Misra S.K. (2021). Ion Exchange Based Approach for Rapid and Selective Pb(II) Removal Using Iron Oxide Decorated Metal Organic Framework Hybrid. J. Environ. Manag..

[14] Jeong C., Ansari M.Z., Hakeem Anwer A., Kim S.-H., Nasar A., Shoeb M., Mashkoor F. (2023). A Review on Metal-Organic Frameworks for the Removal of Hazardous Environmental Contaminants. Sep. Purif. Technol..

[15] Lin G., Zeng B., Li J., Wang Z., Wang S., Hu T., Zhang L. (2023). A Systematic Review of Metal Organic Frameworks Materials for Heavy Metal Removal: Synthesis, Applications and Mechanism. Chem. Eng. J..

[16] Ghosh S.K. (2019). Metal-Organic Frameworks (MOFs) for Environmental Applications.

[17] Khan A., Abu-Zaid B.M., Hussein M.A., Asiri A.M., Azam M. (2019). Metal-Organic Framework Composites—Volume I.

[18] Perera A.A.P.R., Madhushani K.A.U., Kumar A., Gupta R.K. (2023). Metal-Organic Frameworks for Wastewater Treatment: Recent Developments, Challenges, and Future Prospects. Chemosphere.

[19] Dutta S., Let S., Sharma S., Mahato D., Ghosh S.K. (2021). Recognition and Sequestration of Toxic Inorganic Water Pollutants with Hydrolytically Stable Metal-Organic Frameworks. Chem. Rec..

[20] Molavi H., Hakimian A., Shojaei A., Raeiszadeh M. (2018). Selective Dye Adsorption by Highly Water Stable Metal-Organic Framework: Long Term Stability Analysis in Aqueous Media. Appl. Surf. Sci..

[21] Yuan N., Gong X.-R., Han B.-H. (2021). Hydrophobic Fluorous Metal–Organic Framework Nanoadsorbent for Removal of Hazardous Wastes from Water. ACS Appl. Nano Mater..

[22] Kaur H., Devi N., Siwal S.S., Alsanie W.F., Thakur M.K., Thakur V.K. (2023). Metal–Organic Framework-Based Materials for Wastewater Treatment: Superior Adsorbent Materials for the Removal of Hazardous Pollutants. ACS Omega.

[23] Cheng D., Zhao L., Li N., Smith S.J.D., Wu D., Zhang J., Ng D., Wu C., Martinez M.R., Batten M.P. (2019). Aluminum Fumarate MOF/PVDF Hollow Fiber Membrane for Enhancement of Water Flux and Thermal Efficiency in Direct Contact Membrane Distillation. J. Membr. Sci..

[24] Yang F., Efome J.E., Rana D., Matsuura T., Lan C. (2018). Metal–Organic Frameworks Supported on Nanofiber for Desalination by Direct Contact Membrane Distillation. ACS Appl. Mater. Interfaces.

[25] Efome J.E., Rana D., Matsuura T., Yang F., Cong Y., Lan C.Q. (2020). Triple-Layered Nanofibrous Metal–Organic Framework-Based Membranes for Desalination by Direct Contact Membrane Distillation. ACS Sustain. Chem. Eng..

[26] Li H., Liu H., Shi W., Zhang H., Zhou R., Qin X. (2020). Preparation of Hydrophobic Zeolitic Imidazolate Framework-71 (ZIF-71)/PVDF Hollow Fiber Composite Membrane for Membrane Distillation through Dilute Solution Coating. Sep. Purif. Technol..

[27] DeCoste J.B., Peterson G.W., Jasuja H., Glover T.G., Huang Y., Walton K.S. (2013). Stability and Degradation Mechanisms of Metal–Organic Frameworks Containing the Zr_6_O_4_(OH)_4_ Secondary Building Unit. J. Mater. Chem. A.

[28] Rasheed T. (2023). Water Stable MOFs as Emerging Class of Porous Materials for Potential Environmental Applications. Chemosphere.

[29] Antwi-Baah R., Liu H. (2018). Recent Hydrophobic Metal-Organic Frameworks and Their Applications. Materials.

[30] Álvarez J.R., Sánchez-González E., Pérez E., Schneider-Revueltas E., Martínez A., Tejeda-Cruz A., Islas-Jácome A., González-Zamora E., Ibarra I.A. (2017). Structure Stability of HKUST-1 towards Water and Ethanol and Their Effect on Its CO_2_ Capture Properties. Dalton Trans..

[31] Ahmadijokani F., Molavi H., Rezakazemi M., Tajahmadi S., Bahi A., Ko F., Aminabhavi T.M., Li J.-R., Arjmand M. (2022). UiO-66 Metal–Organic Frameworks in Water Treatment: A Critical Review. Prog. Mater. Sci..

[32] Nguyen J.G., Cohen S.M. (2010). Moisture-Resistant and Superhydrophobic Metal−Organic Frameworks Obtained via Postsynthetic Modification. J. Am. Chem. Soc..

[33] Zha Q., Sang X., Liu D., Wang D., Shi G., Ni C. (2019). Modification of Hydrophilic Amine-Functionalized Metal-Organic Frameworks to Hydrophobic for Dye Adsorption. J. Solid State Chem..

[34] Karmakar A., Pombeiro A.J.L. (2019). Recent Advances in Amide Functionalized Metal Organic Frameworks for Heterogeneous Catalytic Applications. Coord. Chem. Rev..

[35] Hintz H., Wuttke S. (2014). Solvent-Free and Time Efficient Postsynthetic Modification of Amino-Tagged Metal–Organic Frameworks with Carboxylic Acid Derivatives. Chem. Mater..

[36] Sinha Ray S., Singh Bakshi H., Dangayach R., Singh R., Deb C.K., Ganesapillai M., Chen S.-S., Purkait M.K. (2020). Recent Developments in Nanomaterials-Modified Membranes for Improved Membrane Distillation Performance. Membranes.

[37] Badertscher M., Bülhmann P., Pretsch E. (2009). Structure Determination of Organic Compounds: Tables of Spectral Data.

[38] Sastre A., Díaz-García M.A., Del Rey B., Dhenaut C., Zyss J., Ledoux I., Agulló-López F., Torres T. (1997). Push−Pull Phthalocyanines: A Hammett Correlation between the Cubic Hyperpolarizability and the Donor–Acceptor Character of the Substituents. J. Phys. Chem. A.

[39] Luan Y., Qi Y., Gao H., Andriamitantsoa R.S., Zheng N., Wang G. (2015). A General Post-Synthetic Modification Approach of Amino-Tagged Metal–Organic Frameworks to Access Efficient Catalysts for the Knoevenagel Condensation Reaction. J. Mater. Chem. A.

[40] Patterson A.L. (1939). The Scherrer Formula for X-ray Particle Size Determination. Phys. Rev..

[41] Moffet R.C., Tivanski A.V., Gilles M.K. Scanning Transmission X-ray Microscopy: Applications in Atmospheric Aerosol Reseach. https://digital.library.unt.edu/ark:/67531/metadc831481/.

[42] Fang X., Wu S., Wu Y., Yang W., Li Y., He J., Hong P., Nie M., Xie C., Wu Z. (2020). High-Efficiency Adsorption of Norfloxacin Using Octahedral UIO-66-NH2 Nanomaterials: Dynamics, Thermodynamics, and Mechanisms. Appl. Surf. Sci..

[43] Abe T., Watanabe A. (2004). X-ray Photoelectron Spectroscopy of Nitrogen Functional Groups in Soil Humic Acids. Soil Sci..

[44] Biesinger M.C., Lau L.W.M., Gerson A.R., Smart R.S.C. (2010). Resolving Surface Chemical States in XPS Analysis of First Row Transition Metals, Oxides and Hydroxides: Sc, Ti, V, Cu and Zn. Appl. Surf. Sci..

[45] Molefe L.Y., Musyoka N.M., Ren J., Langmi H.W., Mathe M., Ndungu P.G. (2019). Polymer-Based Shaping Strategy for Zeolite Templated Carbons (ZTC) and Their Metal Organic Framework (MOF) Composites for Improved Hydrogen Storage Properties. Front. Chem..

[46] Wang B., Lv X.-L., Feng D., Xie L.-H., Zhang J., Li M., Xie Y., Li J.-R., Zhou H.-C. (2016). Highly Stable Zr(IV)-Based Metal–Organic Frameworks for the Detection and Removal of Antibiotics and Organic Explosives in Water. J. Am. Chem. Soc..

[47] Užarević K., Wang T.C., Moon S.-Y., Fidelli A.M., Hupp J.T., Farha O.K., Friščić T. (2016). Mechanochemical and Solvent-Free Assembly of Zirconium-Based Metal–Organic Frameworks. Chem. Commun..

[48] Jayaramulu K., Geyer F., Schneemann A., Kment Š., Otyepka M., Zboril R., Vollmer D., Fischer R.A. (2019). Hydrophobic Metal–Organic Frameworks. Adv. Mater..

[49] Armarego W.L.F. (2017). Purification of Laboratory Chemicals.

[50] Schaate A., Roy P., Godt A., Lippke J., Waltz F., Wiebcke M., Behrens P. (2011). Modulated Synthesis of Zr-Based Metal-Organic Frameworks: From Nano to Single Crystals. Chem. Eur. J..

[51] Rietveld H.M. (1969). A Profile Refinement Method for Nuclear and Magnetic Structures. J. Appl. Crystallogr..

[52] Rodríguez-Carvajal J. FULLPROF: A Program for Rietveld Refinement and Pattern Matching Analysis. Proceedings of the Abstracts of the Satellite Meeting on Powder Diffraction of the XV Congress of the IUCr.

[53] Fairley N., Fernandez V., Richard-Plouet M., Guillot-Deudon C., Walton J., Smith E., Flahaut D., Greiner M., Biesinger M., Tougaard S. (2021). Systematic and Collaborative Approach to Problem Solving Using X-ray Photoelectron Spectroscopy. Appl. Surf. Sci. Adv..

