# Supplementary material for: Novel Hydrophobic Functionalized UiO-66 Series: Synthesis, Characterization, and Evaluation of Their Structural and Physical–Chemical Properties
Source: Int J Mol Sci. 2023 Dec 22;25(1):199. doi: 10.3390/ijms25010199 (PMC10778709; doi:10.3390/ijms25010199)
Supplement: Supplementary file 1 [file ijms-25-00199-s001.zip › ijms-2748533-supplementary.pdf]

Supplementary information

Novel hydrophobic functionalized UiO-66 series: Synthesis, characterization, and evaluation of their structural and physical-chemical properties.

Pilar Narea[1], Iván Brito*[1], Yurieth Quintero[2,3], Esteban Camú[4]

[1] *Departamento de Química, Facultad de Ciencias Básicas, Universidad de Antofagasta, Campus Coloso, Antofagasta 1240000, Chile.*

[2] *Materials Science and Process Engineering PhD Program, Universidad Tecnologica Metropolitana (UTEM), Santiago 8940577, Chile*

[3] *Advanced Mining Technology Center (AMTC), Universidad de Chile, Av. Tupper 2007, 8370451 Santiago, Chile*

[4] *Departamento de Ingeniería Química y Bioprocesos, Facultad de Ingeniería, Pontificia Universidad Católica de Chile, Avenida Vicuña Mackenna 4860, Santiago, Chile*

Correspondence email: ivan.brito@uantof.cl

Page

Table of contents

Figure S1. NMR spectra of UiO66-NH_2

Figure S2. N*1s* HR-XPS spectra of the synthesized compounds: a) UiO66-NH_2, b) (**1**), c) (**2**), d) (**3**) and e) (**4**).

Figure S3. EDX of the synthesized compounds: a) UiO66-NH_2, b) (**1**), c) (**2**), d) (**3**) and e) (**4**).

Figure S4. Qualitative hydrophobicity test of the resulting compounds

Figure S5. Contact angle measurements using sessile drop method for the synthesized compounds. a) 1, b) 2, c) 3 and d) 4.

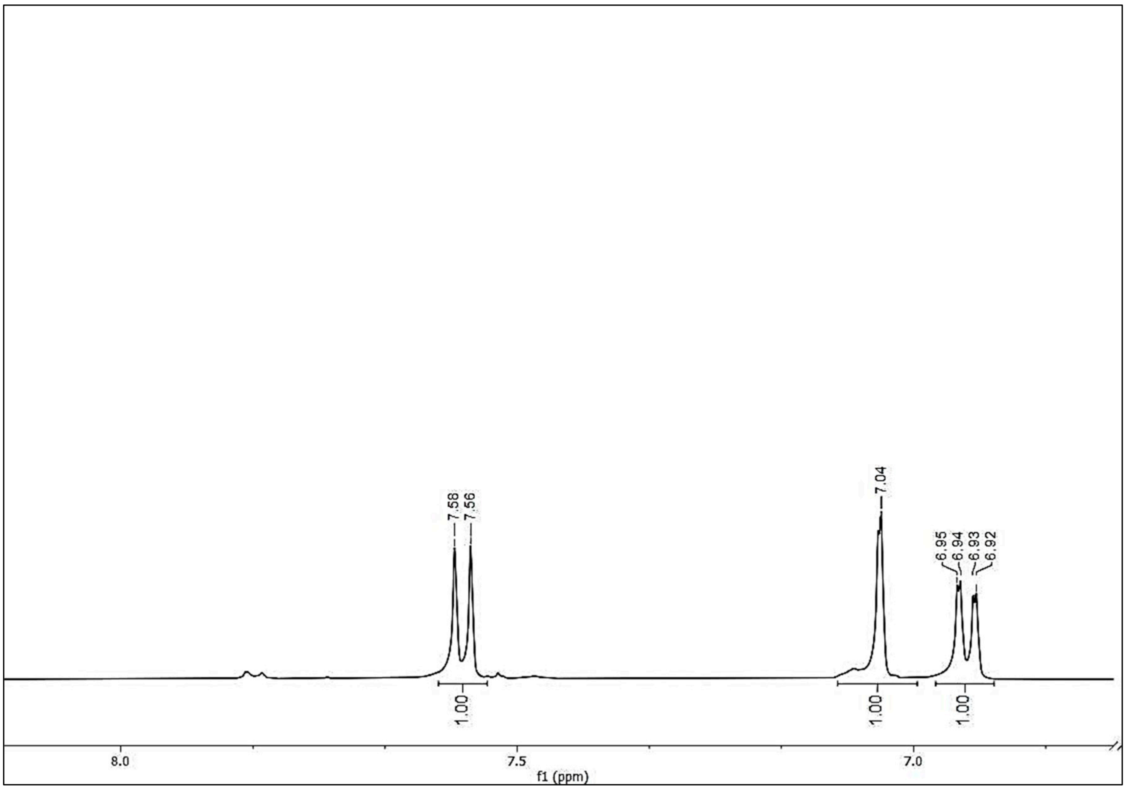

Figure S1. NMR spectra of UiO66-NH_2

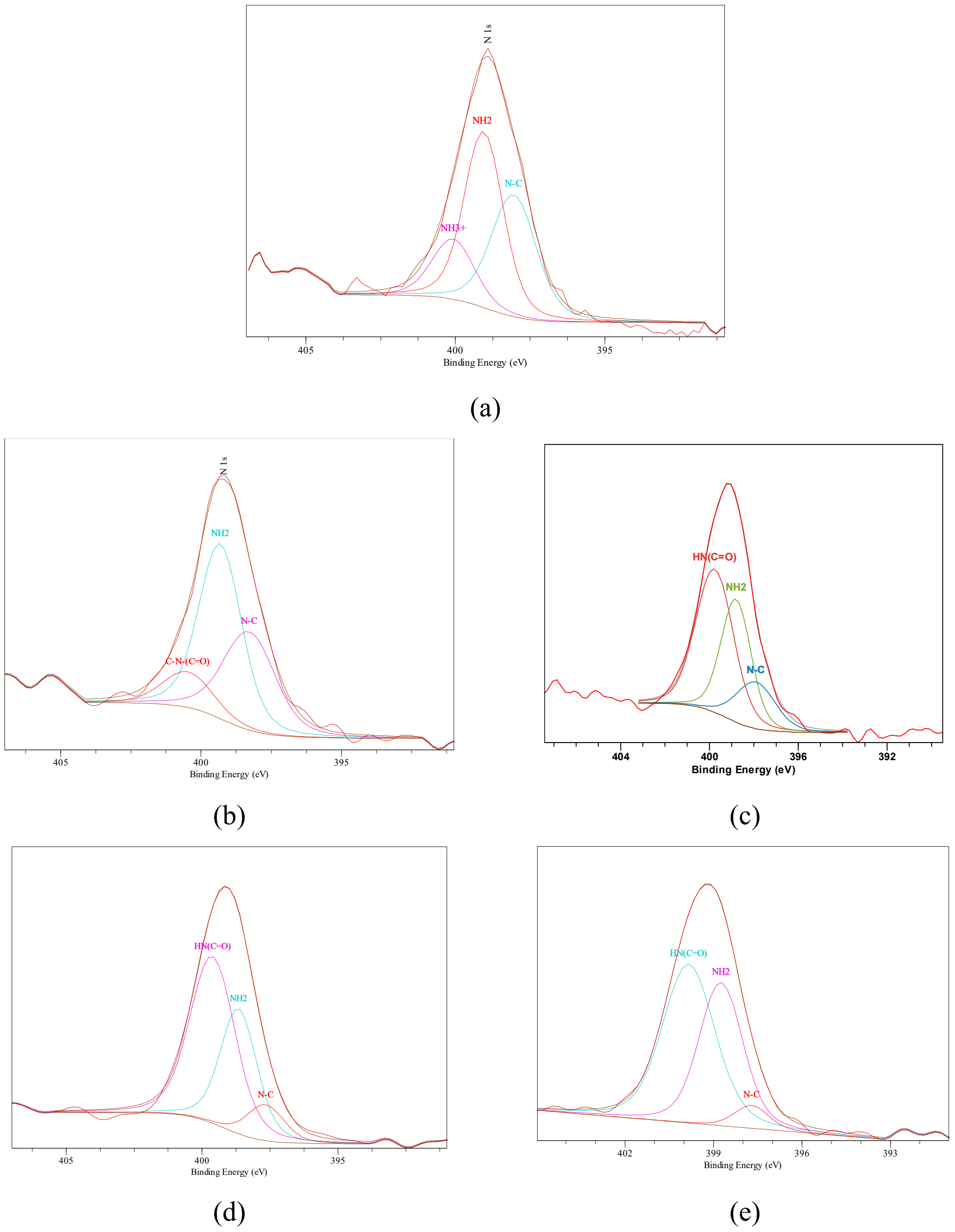

Figure S2. N*1s* HR-XPS spectra of the synthesized compounds: a) UiO66-NH_2, b) (**1**), c) (**2**), d) (**3**) and e) (**4**).

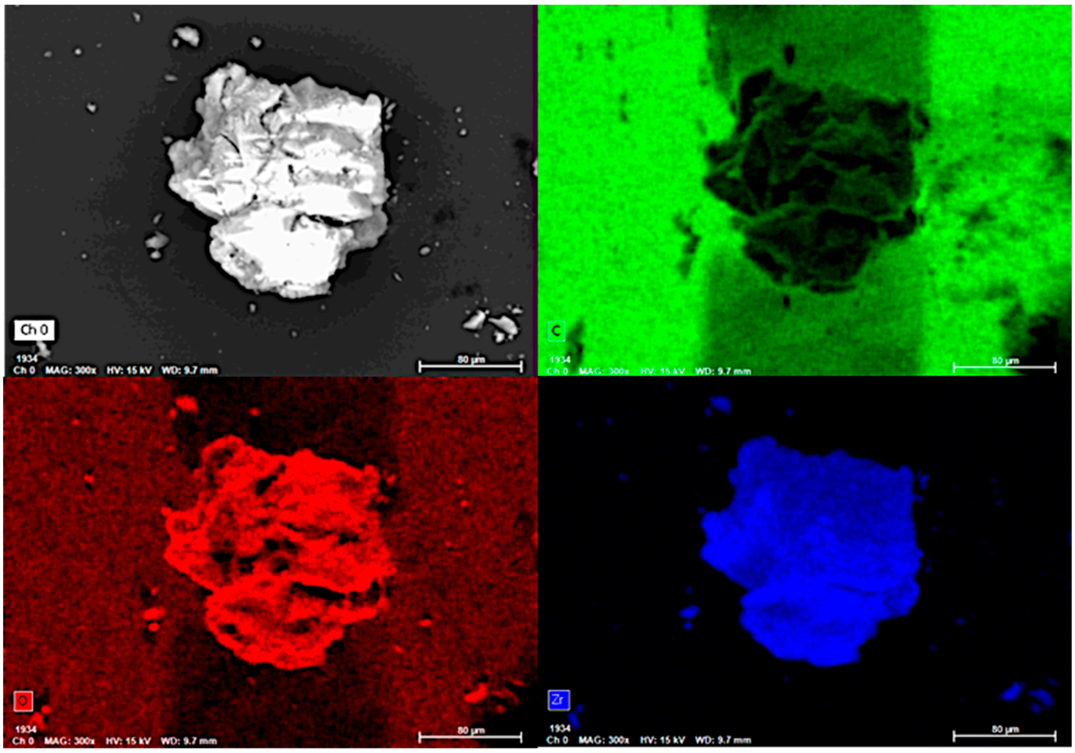

(a)

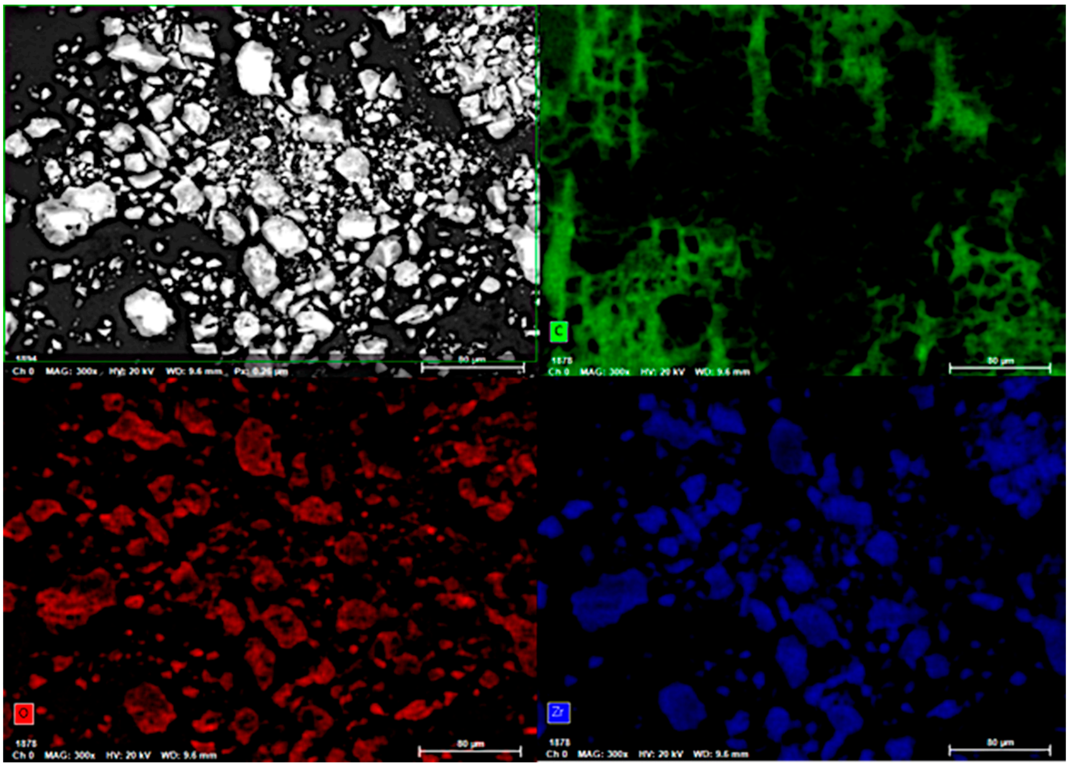

(b)

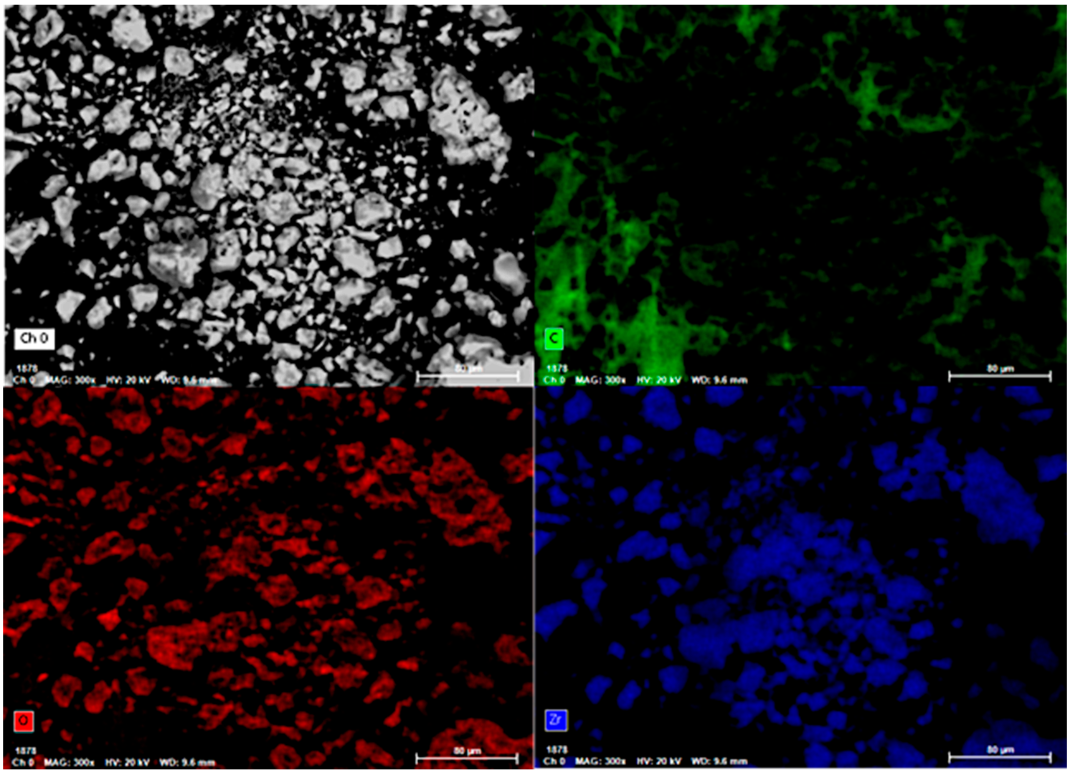

(c)

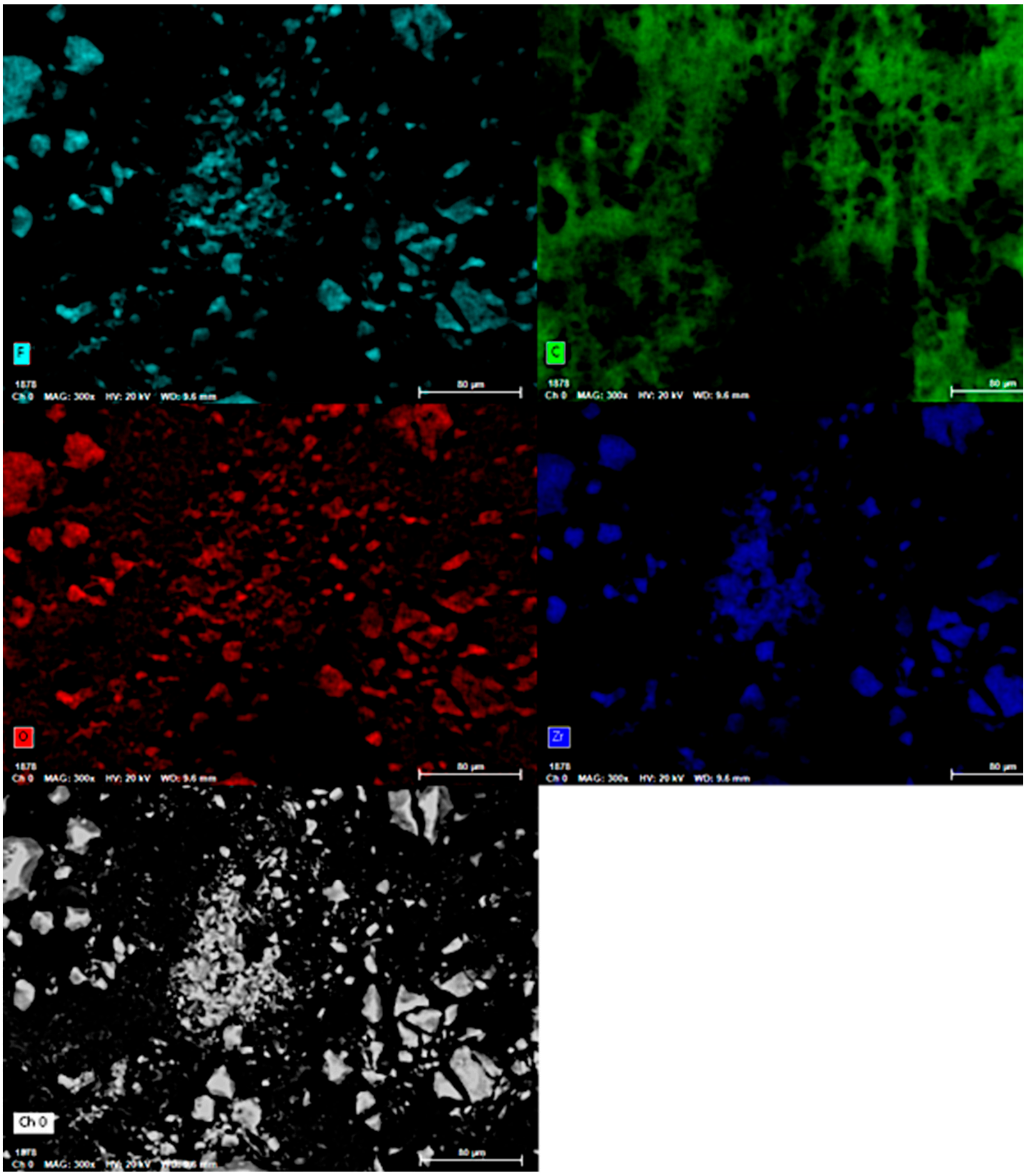

(d)

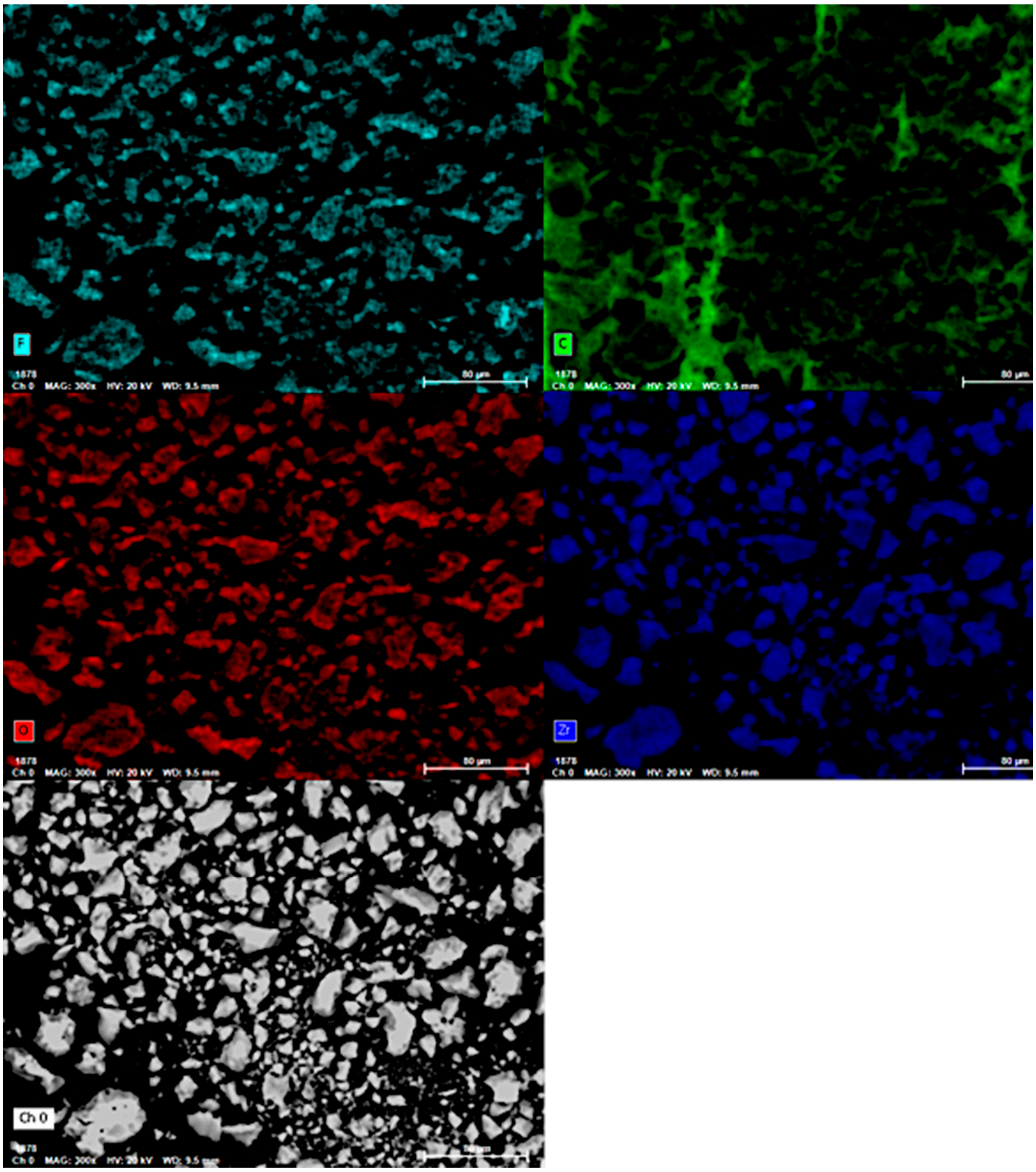

(e)

Figure S3. EDX of the synthesized compounds: a) UiO66-NH_2, b) (**1**), c) (**2**), d) (**3**) and e) (**4**).

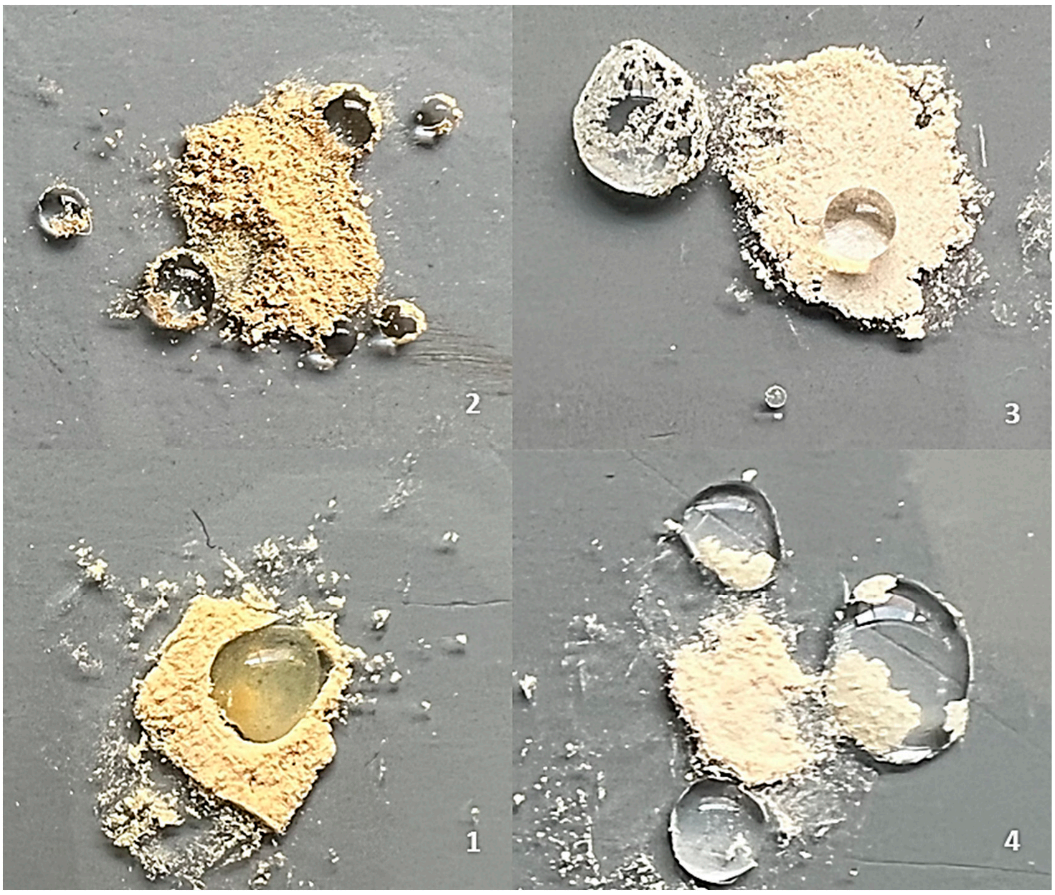

Figure S4. Qualitative hydrophobicity test of the resulting compounds

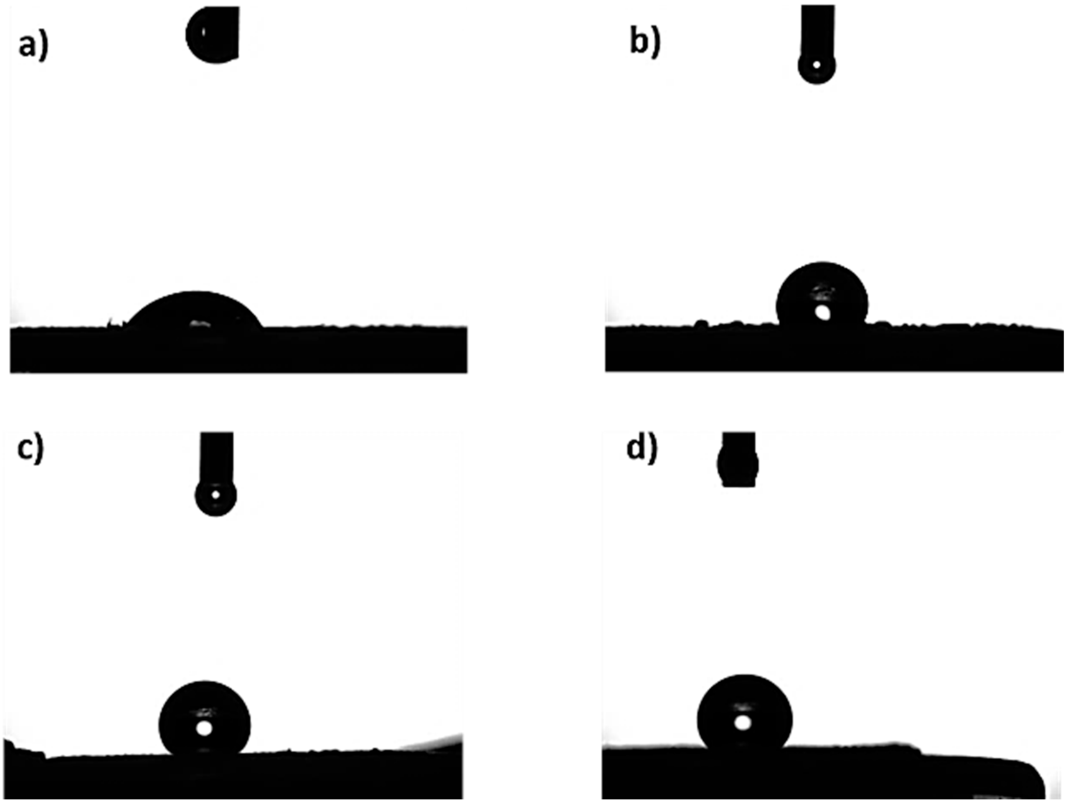

Figure S5. Contact angle measurements using sessile drop method for the synthesized compounds. a) 1, b) 2, c) 3 and d) 4.
